# Beyond Size: Integrating Ultrasonographic Features and FNAB Cytology to Predict Thyroid Malignancy—A Retrospective, Single-Center Study

**DOI:** 10.3390/jcm15020419

**Published:** 2026-01-06

**Authors:** Nihal Güngör Tunç, Cengiz Durucu, Orhan Tunc

**Affiliations:** 1 Department of Otorhinolaryngology, Private Clinics, 27310 Gaziantep, Türkiye; nihalgungor03@yahoo.com; 2 Department of Otorhinolaryngology, Faculty of Medicine, Gaziantep University, 27310 Gaziantep, Türkiye; orhantip@hotmail.com

**Keywords:** thyroid nodule, fine-needle aspiration biopsy, ultrasonography, Bethesda system, TI-RADS, malignancy prediction

## Abstract

**Background/Objectives:** This study aimed to evaluate the relationship between preoperative clinical, ultrasonographic, and cytologic findings and postoperative histopathology in patients with thyroid nodules, and to determine diagnostic factors associated with malignancy. **Materials and Methods:** A retrospective analysis was conducted on 100 patients who underwent thyroid surgery between September 2012 and April 2014. Preoperative data—including clinical examination, thyroid function tests, and high-resolution ultrasonography—were compared with fine-needle aspiration biopsy (FNAB) results and final histopathology. Ultrasonographic features (echogenicity, calcification, vascularity, and margin) were analyzed for their association with malignancy. Statistical tests included chi-square, *t*-test, and correlation analysis (*p* < 0.05 considered significant). **Results:** Among 100 patients (79 females, 21 males; mean age 47.5 ± 13.9 years), 29 (29%) had benign and 71 (71%) malignant histopathology. Malignancy was significantly associated with older age (*p* = 0.025), smaller nodule size (*p* = 0.019), hypoechogenicity (*p* = 0.001), microcalcifications (*p* = 0.014), and irregular margins (*p* = 0.017). FNAB showed a strong correlation with final histopathology (r = 0.65, *p* = 0.001). The overall sensitivity and specificity of FNAB were 25.4% and 82.8%, respectively. **Conclusions:** Hypoechogenicity, microcalcifications, and irregular margins were the most reliable ultrasonographic predictors of malignancy. FNAB remains a highly specific but variably sensitive diagnostic tool, and its accuracy increases when interpreted in conjunction with ultrasonographic findings. Integrating cytology with structured imaging systems such as ACR TI-RADS and Bethesda classification enhances diagnostic precision in thyroid nodule evaluation.

## 1. Introduction

Thyroid nodules are among the most frequently encountered endocrine abnormalities, with prevalence rising in step with aging populations and the widespread use of high-resolution ultrasonography (US). Contemporary guidelines emphasize that, while most nodules are benign, a crucial minority harbor differentiated thyroid cancer, making risk stratification and judicious use of biopsy central to care [1]. US has become the cornerstone for initial evaluation, complemented by thyroid-stimulating hormone (TSH) testing and selective scintigraphy, but imaging alone cannot definitively distinguish all benign from malignant lesions [2,3]. Fine-needle aspiration (FNA) under US guidance therefore remains the pivotal diagnostic test in most pathways because it reduces unnecessary surgery and directs the extent of thyroidectomy when malignancy is likely [4,5,6,7,8].

Over the past decade, structured reporting systems have substantially improved consistency in risk estimation and decision-making. The American College of Radiology Thyroid Imaging Reporting and Data System (ACR TI-RADS) introduced a point-based lexicon that stratifies malignancy risk from TR1 to TR5 and links categories to evidence-based thresholds for FNA or follow-up, reducing avoidable biopsies without compromising cancer detection [9,10,11,12]. Parallel advances in cytopathology culminated in the third edition of The Bethesda System for Reporting Thyroid Cytopathology (TBSRTC), which refined diagnostic categories, updated implied risks of malignancy, and aligned reporting with contemporary histopathology and molecular insights [4]. These frameworks, alongside the 2022/2023 fifth-edition World Health Organization (WHO) classification of thyroid tumors, which clarified entities and biological behavior, collectively tighten the interface between imaging impressions, cytologic categories, and histologic outcomes [13,14].

Although the vast majority of thyroid fine-needle aspiration cytologies classified as Bethesda II (benign) are associated with a low risk of malignancy, recent data demonstrate that even this category may carry a measurable cancer risk. Mulita et al. reported a malignancy rate of 1.5% among 522 surgically managed Bethesda II nodules [15]. Conversely, Bethesda III (AUS/FLUS) nodules show considerably higher variability, with pooled malignancy rates ranging between 18% and 20% in contemporary cohorts, as demonstrated in a 2022 study by Mulita et al. [16]. Incorporating these epidemiological data highlights the clinical importance of integrating ultrasonographic features with cytology to improve diagnostic accuracy across intermediate-risk categories.

Despite these standardized frameworks, real-world performance varies across institutions and case-mix. Reported sensitivity and specificity of US-guided FNA are generally high, yet they depend on operator expertise, nodule characteristics (size, composition, calcifications), and the prevalence of indeterminate cytology (Bethesda III/IV) [17,18,19]. Moreover, the relationship between nodule size and malignancy risk remains nuanced: while larger nodules are more likely to be referred for surgery, malignancy can concentrate within smaller, highly suspicious lesions per TI-RADS features [20,21]. Institutional audits therefore remain critical to validate pathway performance against local epidemiology, imaging practice, and cytopathology expertise.

Within this context, previous studies in the literature have systematically compared preoperative clinical examination, laboratory tests, ultrasonographic findings, and FNA results with postoperative histopathology in surgical cohorts, highlighting meaningful correlations and underscoring the need to interpret FNA findings alongside imaging and clinical parameters to optimize diagnostic accuracy [22,23,24,25]. These studies also demonstrated that diagnostic yield is context-dependent and may vary across populations, reinforcing the importance of continuous quality evaluation throughout the diagnostic process.

Therefore, the primary objective of this study was to determine which preoperative ultrasonographic features and fine-needle aspiration biopsy (FNAB) cytology findings are most strongly associated with thyroid malignancy when compared with postoperative histopathology. A secondary objective was to evaluate the diagnostic performance of FNAB across Bethesda categories and to explore how ultrasonographic characteristics—including echogenicity, calcifications, margins, and nodule size—interact with cytologic classification in predicting malignancy. By integrating these parameters, this study aimed to provide a clearer understanding of how preoperative imaging and cytology can be combined to optimize risk stratification in patients undergoing surgery for thyroid nodules.

## 2. Materials and Methods

### 2.1. Study Design and Patient Selection

This retrospective study was conducted at the Department of Otorhinolaryngology, Faculty of Medicine, Gaziantep University, between September 2012 and April 2014, following approval by the Gaziantep University Local Ethics Committee (Approval No: 10.02.2014/69, dated 10 February 2014). The study adhered to the principles of the Declaration of Helsinki and current institutional research guidelines. Medical records of 100 patients who presented with thyroid nodules and subsequently underwent thyroid surgery were reviewed retrospectively. Patients with recurrent disease, those who had undergone completion thyroidectomy, or those lacking fine-needle aspiration biopsy (FNAB) results were excluded from the study. In cases where multiple FNABs were performed, only the most recent biopsy result was considered.

Because this was a retrospective study, no a priori sample size calculation or power analysis was performed. The sample size was determined by the total number of patients who met the inclusion criteria and underwent thyroid surgery during the predefined study period (September 2012–April 2014). Retrospective cytology–histology correlation studies in the thyroid literature commonly include similar or smaller sample sizes, and a cohort of 100 patients provides adequate statistical power for comparative analyses of categorical ultrasonographic and cytologic variables. In addition, certain clinical variables—such as smoking status, prior malignancy, history of radiation exposure, and hematologic disorders—were not uniformly available in the medical records and therefore could not be included in the comparative analyses.

### 2.2. Preoperative Evaluation

All patients underwent a comprehensive preoperative evaluation consisting of detailed physical examination, measurement of thyroid function parameters (serum thyroid-stimulating hormone [TSH], free thyroxine [FT4], and free triiodothyronine [FT3]), and high-resolution ultrasonography (US) of the thyroid gland. Each detected nodule was characterized sonographically with respect to its number (solitary or multiple), maximal diameter (0–10 mm, 11–20 mm, 21–30 mm, 31–40 mm, or >41 mm), echogenicity (hypoechoic, hyperechoic, or mixed), internal structure (solid, cystic, or mixed), pattern of calcification (micro- or macro-calcifications), Doppler vascularity (hypovascular or hypervascular), and margin definition (regular or irregular). Nodule size on physical examination refers to palpation-based size estimation documented during the initial ENT evaluation, whereas nodule size on ultrasonography represents precise measurements obtained with high-resolution US imaging.

Nodules selected for fine-needle aspiration biopsy (FNAB) were preferentially those demonstrating sonographic features associated with malignancy—particularly hypoechogenicity, irregular or infiltrative borders, and the presence of microcalcifications—irrespective of size. In mixed solid-cystic nodules, aspiration was directed toward the solid component to ensure maximal cellular yield and representative cytologic sampling. All ultrasonographic examinations were performed by a single experienced radiologist who was not involved as an author in this study. This ensured consistency in imaging assessment and eliminated inter-observer variability. The nodule size categories (0–10 mm, 11–20 mm, 21–30 mm, 31–40 mm, and >40 mm) reflect the fixed measurement intervals used in the institutional ultrasonography reporting system during 2012–2014. These predefined categories were adopted without modification to preserve accuracy of the original records, although we acknowledge that TNM-based thresholds (10 mm and 20 mm) may be more clinically meaningful.

### 2.3. Fine-Needle Aspiration Biopsy (FNAB) and Cytology Classification

Ultrasound-guided FNAB was performed by experienced radiologists under strict aseptic conditions using a 25-gauge needle attached to a 10 mL syringe. Each procedure was directed toward the most suspicious area of the target nodule, with multiple passes made when necessary to obtain adequate cellular material. The aspirated samples were immediately smeared onto glass slides, air-dried for May–Grünwald–Giemsa staining, and alcohol-fixed for Papanicolaou staining. Cytologic evaluation was conducted by an experienced cytopathologist following the criteria of the Bethesda System for Reporting Thyroid Cytopathology [26]. Based on these criteria, all cytologic results were classified into five diagnostic categories: benign; atypia of undetermined significance or follicular lesion of undetermined significance (AUS/FLUS); follicular neoplasm or suspicious for follicular neoplasm; suspicious for malignancy; and malignant. This standardized classification ensured comparability with contemporary literature and provided a structured basis for correlating preoperative cytology with postoperative histopathology findings.

### 2.4. Histopathological Evaluation

**Figure 1:**
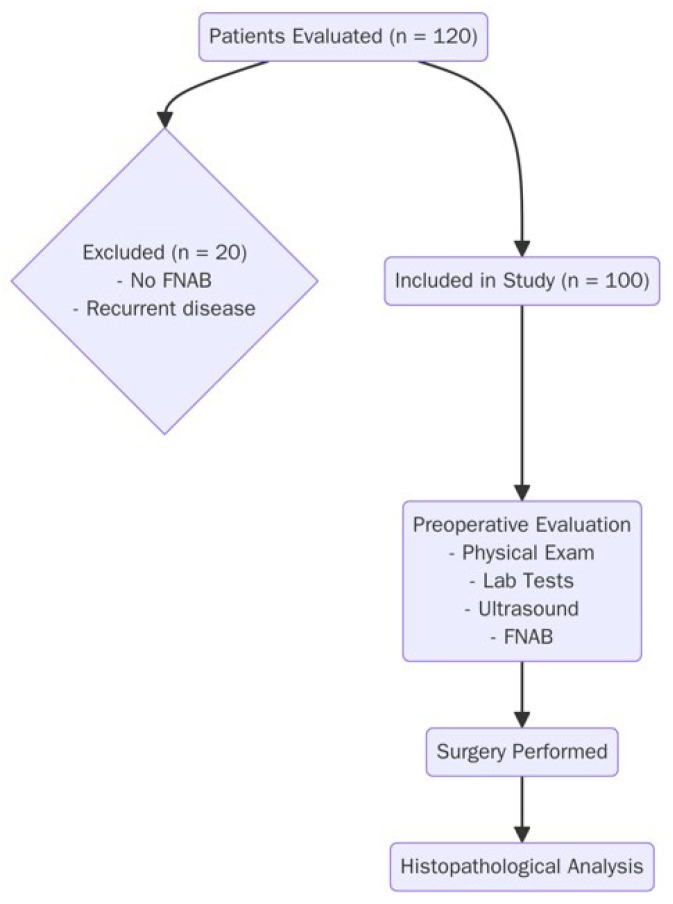
Flowchart of the study.

Postoperative pathology reports were reviewed to establish definitive diagnoses. Cases reported as papillary carcinoma, follicular carcinoma, medullary carcinoma, Hurthle cell carcinoma, or anaplastic carcinoma were classified as malignant. Diagnoses of nodular or multinodular goiter, adenoma, Hurthle cell adenoma, or hyperplasia were classified as benign (Figure 1).

### 2.5. Statistical Analysis

Statistical analyses were carried out using SPSS for Windows, version 25.0 (IBM Corp., Armonk, NY, USA). The Kolmogorov–Smirnov test was used to determine whether continuous variables followed a normal distribution. Data were expressed as mean ± standard deviation (SD) for continuous variables and as counts and percentages for categorical variables. For group comparisons, the student’s *t*-test was applied to normally distributed variables, whereas the Mann–Whitney U test was used for non-parametric data. Associations between categorical variables were assessed using the chi-square (χ^2^) test. Diagnostic performance was quantified through calculation of sensitivity [true positive/(true positive + false negative)], specificity [true negative/(true negative + false positive)], positive predictive value [true positive/(true positive + false positive)], and negative predictive value [true negative/(true negative + false negative)] in accordance with standard definitions. All statistical tests were two-tailed, and a *p*-value < 0.05 was considered statistically significant.

## 3. Results

A total of 100 patients (79 females and 21 males) who underwent thyroid surgery for nodular disease between September 2012 and April 2014 were evaluated. The mean age of the study population was 47.50 ± 13.90 years, ranging from 14 to 84 years. The mean age was significantly higher in the malignant group compared to the benign group (49.54 ± 13.59 vs. 42.59 ± 14.39 years, *p* = 0.025). There was no significant relationship between sex and malignancy (*p* = 0.258).

Based on physical examination, 40% of patients had a single thyroid nodule and 60% had multiple nodules. Nodule sizes ranged from 0 to >40 mm and were significantly associated with malignancy (*p* = 0.034). Malignant nodules were more common in smaller size categories, whereas benign lesions predominated in larger nodules.

Postoperative histopathological evaluation revealed 29 benign (29%) and 71 malignant (71%) cases. Among benign lesions, 27 (27%) were multinodular goiter and 2 (2%) were follicular adenoma. Among malignant cases, papillary carcinoma was the most frequent diagnosis, observed in 67 patients (67%), followed by follicular carcinoma (2%) and medullary carcinoma (2%) (Table 1).

According to Table 2, ultrasonographic findings were assessed in relation to malignancy. Among 100 patients, 40% had a single thyroid nodule and 60% had multiple nodules. The number of nodules was not significantly associated with malignancy (*p* = 0.126). Nodule size measured on ultrasonography ranged from less than 10 mm to greater than 40 mm and showed a statistically significant relationship with malignancy (*p* = 0.019). Smaller nodules (0–30 mm) were more frequently malignant, whereas larger nodules were predominantly benign (Table 2).

Evaluation of ultrasonographic morphological features revealed significant associations between multiple imaging parameters and malignancy. Echogenicity was strongly related to malignant pathology (*p* = 0.001): hypoechoic nodules constituted 57% of all lesions and showed a markedly higher proportion of malignancy (67.6%), whereas hyperechoic nodules were more commonly benign (58.6%). Internal structure also demonstrated a significant relationship with malignancy (*p* = 0.001). Solid nodules were predominant among malignant cases (80.3%), while cystic nodules were more frequent in benign lesions (20.7%).

Calcification pattern was significantly associated with malignancy (*p* = 0.014). Of the 100 nodules, 61% showed no calcification, 22% exhibited microcalcifications, and 17% had macrocalcifications. Microcalcification was particularly indicative of malignancy (28.2% vs. 6.9% in benign lesions).

Vascularity pattern did not exhibit a significant correlation with malignancy (*p* = 0.240), with hypovascularity observed in 46% and hypervascularity in 54% of nodules. Margin characteristics, however, demonstrated a strong association with malignant pathology (*p* = 0.017). Irregular margins were present in 27% of all nodules and were considerably more common in malignant cases (33.8%) than in benign ones (10.3%) (Table 3).

Odds ratio analysis demonstrated that hypoechogenicity (OR 4.64), microcalcifications (OR 5.29), and irregular margins (OR 4.43) were the strongest ultrasonographic predictors of malignancy. Conversely, hyperechogenicity (OR 0.17), cystic structure (OR 0.49), and regular margins (OR 0.23) were associated with a lower likelihood of malignancy (Supplementary Materials Table S1).

**Figure 2:**
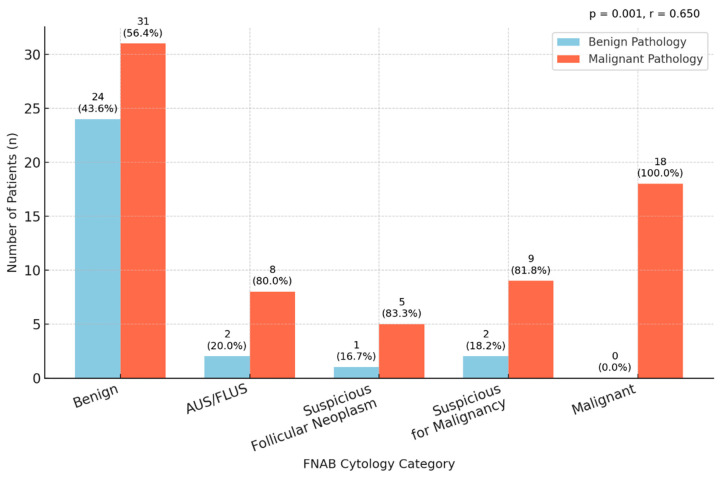
Correlation Between FNAB Cytology and Final Histopathology.

Fine-needle aspiration biopsy (FNAB) cytology findings were compared with final histopathological diagnoses. Among 55 patients classified as benign on FNAB, 31 (43.7%) were confirmed as malignant on histopathology, while 24 (82.8%) remained benign. In the AUS/FLUS category (n = 10), 8 cases (80.0%) were malignant and 2 (20.0%) were benign. Of the 6 cases suspicious for follicular neoplasm, 5 (83.3%) were malignant. In the group suspicious for malignancy (n = 11), 9 (81.8%) were malignant and 2 (18.2%) were benign. All 18 cases reported as malignant on FNAB were confirmed as malignant postoperatively. There was a strong positive correlation between FNAB cytology and final histopathology results (r = 0.650, *p* = 0.001) (Table 4 and Figure 2).

The proportion of malignancy across FNAB diagnostic categories was evaluated. Among patients with benign cytology (n = 55), 31 (56.4%) were confirmed as malignant on final histopathology. In the AUS/FLUS category (n = 10), 8 cases (80.0%) were malignant. For lesions suspicious for follicular neoplasm (n = 6), malignancy was confirmed in 5 cases (83.3%). In the group suspicious for malignancy (n = 11), 9 cases (81.8%) were malignant. All 18 cases (100.0%) reported as malignant on FNAB were verified as malignant on histopathological examination. The difference in malignancy proportions across FNAB categories was statistically significant (*p* = 0.001) (Table 5).

## 4. Discussion

This study evaluated clinical, ultrasonographic, and cytologic factors associated with thyroid malignancy in a surgical cohort and compared preoperative assessments with histopathology. Three result patterns were prominent. First, US morphology—not merely nodule count—tracked with malignancy: hypoechogenicity, microcalcifications, and irregular margins were each significantly associated with cancer, whereas vascularity patterns were not. Second, smaller nodules carried proportionally higher malignancy rates than larger ones within our operated sample. Third, cytology and histology showed a strong monotonic relationship across Bethesda-aligned categories, with malignancy proportions rising from “benign” to “malignant” cytology and a robust overall correlation (r = 0.650). These observations align with contemporary risk-stratification frameworks and recent multi-study evidence, while also reflecting the selection features of an all-surgery cohort.

Findings on US features run parallel to the factors which structured systems like ACR TI-RADS encode as high-risk attributes. Tessler et al. formalized a point-based lexicon in which hypoechogenicity, microcalcifications, and non-smooth margins drive category upgrades and biopsy recommendations [2]. Reviews and meta-analyses synthesized since then consistently report the same features as the most predictive of malignancy: hypoechogenicity and punctate echogenic foci (microcalcifications) have among the highest likelihood ratios, while margin irregularity contributes additional risk even in small nodules [20,27]. Our results reproduce this triad and, like others, found vascularity to be an unreliable discriminator, a limitation also noted in recent appraisals of US predictors [2,28]. That concordance supports the continued emphasis on TI-RADS-aligned morphology over Doppler patterns in triage.

Regarding nodule size, our surgical series showed relatively higher malignancy proportions in smaller size strata and lower proportions in the largest lesions. Although size frequently informs biopsy thresholds, modern guidance underscores that morphologic risk outweighs size per se, especially for TR4–TR5 nodules where biopsy is advised at relatively small diameters [2,29]. Contemporary cohorts likewise report that cancer is not confined to large nodules; some even describe peak malignancy prevalence around 1–2 cm when high-risk features are present [28,30]. Taken together, these data and our results support practice patterns that prioritize morphology-based thresholds over a size-only paradigm.

The cytology–histology gradient we observed mirrors the updated risk-of-malignancy (ROM) framework of the 2023 Bethesda revision. Ali et al. recalibrated ROMs and harmonized terminology with the 2022 WHO classification, clarifying expectations for AUS/FLUS, follicular-patterned lesions, and “suspicious for malignancy” categories [4,13,14]. In our cohort, malignancy proportions rose stepwise from benign → AUS/FLUS → follicular neoplasm/suspicious → suspicious for malignancy → malignant, echoing the Bethesda continuum. Large pooled evidence supports this overall alignment: a 2022 JAMA Surgery meta-analysis (>16,000 patients) reported high accuracy of thyroid fine-needle biopsy overall, with sensitivity ~86% and specificity ~71%, acknowledging inter-study heterogeneity tied to case-mix and reference standards [5]. Single-center and multi-center studies published since 2023 similarly confirm strong cytology–histology agreement—particularly at the extremes of the Bethesda spectrum—while highlighting variability in intermediate categories where imaging context and, increasingly, molecular testing can refine decisions [9,22,24,25]. Our moderate-to-strong correlation is consistent with that literature and with integrative approaches that read TI-RADS and Bethesda in tandem to improve calibration of malignancy risk [2,28].

These results also intersect with system-level developments. The WHO 5th edition re-definitions (e.g., NIFTP boundaries, grading refinements) and Bethesda 2023 terminology updates aim to reduce both over- and under-calling of malignancy, thereby improving alignment between cytology labels and histologic truth [8,9]. Meanwhile, comparative analyses of TI-RADS families (ACR, EU-, K-TIRADS) show broadly similar discrimination but different operating points, reinforcing the need to validate thresholds in local practice and report outcomes transparently [31,32]. Our data—drawn from an operated cohort—underscore that observed malignancy proportions are context-dependent (enriched relative to general clinics) and should be interpreted as performance within a surgical pathway rather than population screening.

Compared with previous studies, this work provides three key distinctions. First, unlike earlier analyses such as Osseis et al. and Aliyev et al. [22,23], which focused mainly on cytology–histology concordance, this study simultaneously evaluated detailed ultrasonographic features and FNAB results, offering a broader view of diagnostic performance. Second, because the cohort predates formal ACR TI-RADS and Bethesda 2023 updates, it reflects pre-guideline diagnostic patterns, serving as a baseline for comparison with modern structured systems reported by Eissa et al. and Mehanna et al. [17,24]. Third, this study quantified the FNAB–histology relationship using a correlation coefficient (r = 0.650), whereas most prior reports relied only on categorical agreement. This numerical approach underscores the graded malignancy risk across Bethesda categories, consistent with recent recalibrations by Ali et al. [4].

The relatively low sensitivity of FNAB observed in our cohort requires careful interpretation. Because this was a retrospective surgical series, the patient population was inherently enriched with nodules selected for surgery due to suspicious sonographic characteristics, clinical concern, or indeterminate cytology. This selection bias is well known to inflate the proportion of false-negative FNAB results and thereby reduce calculated sensitivity in comparison with population-based cohorts or prospective screening studies. Moreover, all cytology samples were obtained between 2012 and 2014, before the implementation of the revised Bethesda 2017 and 2023 criteria and before modern quality enhancements such as on-site adequacy assessment, both of which are associated with improved diagnostic yield in contemporary practice. Operator- and sampling-dependent variability, especially in mixed or predominantly cystic nodules, may also have contributed to the higher malignancy rate in the ‘benign’ cytology group. These factors collectively explain why FNAB sensitivity in this study appears lower than the 70–90% range typically reported in the literature.

### Limitations of the Study

This study has some limitations. The retrospective, single-center design inherently limits generalizability and introduces potential selection bias. The inclusion of only surgical cases may have caused spectrum bias, as patients undergoing surgery typically represent a higher-risk population, leading to an overestimation of malignancy rates. The study period also predates the formal implementation of standardized systems such as ACR TI-RADS and the availability of contemporary molecular adjuncts, which restricts direct comparison with current practice frameworks. Furthermore, inter-observer reproducibility for ultrasonographic and cytologic interpretations was not assessed, precluding evaluation of diagnostic consistency across readers. These limitations may have influenced absolute malignancy proportions—for instance, the apparent increase in malignancy among smaller nodules likely reflects surgical selection—yet they are unlikely to alter the overall direction of associations, which remain consistent with multi-study evidence.

Additionally, important clinical characteristics associated with thyroid cancer risk—such as smoking history, previous malignancy, external neck irradiation, and hematologic disorders like leukemia—were not consistently documented in patient charts, limiting their inclusion and potentially underestimating their influence on malignancy risk.

Despite these constraints, the study possesses notable strengths. It provides a direct head-to-head comparison between targeted ultrasonographic features, FNAB cytology, and postoperative histopathology, employing standardized diagnostic categories that align with contemporary frameworks such as the Bethesda System and ACR TI-RADS. This integrative approach enhances the clinical relevance of the findings and supports the continuing validation of preoperative diagnostic tools in thyroid nodule assessment.

Implications for practice are straightforward. First, US morphology—hypoechogenicity, microcalcifications, and irregular margins—should remain the primary gatekeeper for biopsy and follow-up intensity. Second, clinicians should resist size-only heuristics; small, high-risk nodules warrant FNAB, while large, low-risk nodules may be surveilled when guidelines permit. Third, cytology should be interpreted in concert with standardized imaging risk and, where available, molecular testing for indeterminate categories, in keeping with Bethesda 2023 and WHO 2022. Future work at our institution will prospectively apply ACR TI-RADS scoring and Bethesda-3rd-edition reporting, including inter-reader studies and the selective use of molecular assays, to benchmark outcomes against current ROMs and optimize local thresholds.

## 5. Conclusions

In conclusion, this study demonstrates that specific ultrasonographic features—particularly hypoechogenicity, microcalcifications, and irregular margins—are strongly associated with thyroid malignancy. Fine-needle aspiration biopsy showed a robust alignment with final histopathology across Bethesda categories, despite lower sensitivity within this surgical cohort. Integrating ultrasonographic morphology with cytologic classification enhances malignancy prediction and supports more accurate preoperative risk stratification. These results reinforce the importance of combining imaging and cytology when evaluating thyroid nodules.

## Figures and Tables

**Table 1:** Baseline demographic and clinical characteristics of patients and their association with malignancy.

Variable	Benign (n = 29)	Malignant (n = 71)	Total (n = 100)	*p*-Value
**Age (years, Mean ± SD)**	42.59 ± 14.39	49.54 ± 13.59	47.50 ± 13.90	0.025
**Sex**	**n (%)**	**n (%)**	**n (%)**	0.258
Female	25 (86.2%)	54 (76.1%)	79 (79.0%)	
Male	4 (13.8%)	17 (23.9%)	21 (21.0%)	
Nodule size on physical examination (mm) *				0.034
0–10	3 (10.3%)	11 (15.5%)	14 (14.0%)	
11–20	5 (17.2%)	24 (33.8%)	29 (29.0%)	
21–30	7 (24.1%)	20 (28.2%)	27 (27.0%)	
31–40	10 (34.5%)	6 (8.5%)	16 (16.0%)	
>40	4 (13.8%)	10 (14.1%)	14 (14.0%)	
**Postoperative Histopathological Diagnosis**				—
Multinodular goiter	27 (27.0%)	—	27 (27.0%)	
Follicular adenoma	2 (2.0%)	—	2 (2.0%)	
Papillary carcinoma	—	67 (67.0%)	67 (67.0%)	
Follicular carcinoma	—	2 (2.0%)	2 (2.0%)	
Medullary carcinoma	—	2 (2.0%)	2 (2.0%)	
Total benign/malignant	29 (29.0%)	71 (71.0%)	100 (100%)	

* Nodule size on physical examination represents palpation-based estimation during ENT assessment. Additional risk factors such as smoking history, previous malignancy, and radiation exposure were not consistently available in the medical records and were therefore excluded from comparative analysis.

**Table 2:** Association between ultrasonographic nodule characteristics and malignancy.

Ultrasonographic Parameter	Benign (n = 29)	Malignant (n = 71)	Total (n = 100)	*p*-Value
	n (%)	n (%)	n (%)	
**Number of nodules**				0.126
Single	15 (51.7%)	25 (35.2%)	40 (40.0%)	
Multiple	14 (48.3%)	46 (64.8%)	60 (60.0%)	
Nodule size on ultrasonography (mm) *				0.019
0–10 mm	1 (3.4%)	10 (14.1%)	11 (11.0%)	
11–20 mm	6 (20.7%)	20 (28.2%)	26 (26.0%)	
21–30 mm	5 (17.2%)	22 (31.0%)	27 (27.0%)	
31–40 mm	11 (37.9%)	8 (11.3%)	19 (19.0%)	
>40 mm	6 (20.7%)	11 (15.5%)	17 (17.0%)	

* Nodule size on ultrasonography represents the largest diameter measured by high-resolution ultrasound imaging.

**Table 3:** Association between ultrasonographic morphological features of thyroid nodules and malignancy.

Ultrasonographic Feature	Benign (n = 29)	Malignant (n = 71)	Total (n = 100)	*p*-Value
	n (%)	n (%)	n (%)	
**Echogenicity**				0.001
Hypoechoic	9 (31.0%)	48 (67.6%)	57 (57.0%)	
Hyperechoic	17 (58.6%)	14 (19.7%)	31 (31.0%)	
Mixed Echogenicity	3 (10.3%)	9 (12.7%)	12 (12.0%)	
**Internal Structure**				0.001
Solid	20 (69.0%)	57 (80.3%)	77 (77.0%)	
Cystic	6 (20.7%)	8 (11.3%)	14 (14.0%)	
Mixed	3 (10.3%)	6 (8.4%)	9 (9.0%)	
**Calcification Pattern**				0.014
None	24 (82.8%)	37 (52.1%)	61 (61.0%)	
Microcalcification	2 (6.9%)	20 (28.2%)	22 (22.0%)	
Macrocalcification	3 (10.3%)	14 (19.7%)	17 (17.0%)	
**Vascularity Pattern**				0.240
Hypovascular	16 (55.2%)	30 (42.3%)	46 (46.0%)	
Hypervascular	13 (44.8%)	41 (57.7%)	54 (54.0%)	
**Margin Characteristics**				0.017
Regular	26 (89.7%)	47 (66.2%)	73 (73.0%)	
Irregular	3 (10.3%)	24 (33.8%)	27 (27.0%)	

**Table 4:** Correlation between FNAB cytology (Bethesda classification) and final histopathology.

FNAB Result	Benign Pathology n (%)	Malignant Pathology n (%)	Total n (%)	r and *p*-Value
Benign (Bethesda II)	24 (82.8%)	31 (43.7%)	55 (55.0%)	
AUS/FLUS (Bethesda III)	2 (6.9%)	8 (11.3%)	10 (10.0%)	
Follicular Neoplasm Susp. (Bethesda IV)	1 (3.4%)	5 (7.0%)	6 (6.0%)	
Suspicious for Malignancy (Bethesda V)	2 (6.9%)	9 (12.7%)	11 (11.0%)	
Malignant (Bethesda VI)	0 (0%)	18 (25.4%)	18 (18.0%)	
Total	29 (100%)	71 (100%)	100 (100%)	r: 0.650, 0.001

r: correlation coefficient.

**Table 5:** Proportion of malignancy across FNAB categories (with Bethesda classification).

FNAB Result	Benign Pathology n (%)	Malignant Pathology n (%)	Total n (%)	Proportion of Malignancy (%)	*p*-Value
Benign (Bethesda II)	24 (43.6%)	31 (56.4%)	55	56.4	
AUS/FLUS (Bethesda III)	2 (20.0%)	8 (80.0%)	10	80.0	
Suspicious for Follicular Neoplasm (Bethesda IV)	1 (16.7%)	5 (83.3%)	6	83.3	
Suspicious for Malignancy (Bethesda V)	2 (18.2%)	9 (81.8%)	11	81.8	
Malignant (Bethesda VI)	0 (0%)	18 (100.0%)	18	100.0	
Total	29 (29.0%)	71 (71.0%)	100	—	0.001

## Data Availability

Data is available upon request to the corresponding author.

## Supplementary Materials

The following supporting information can be downloaded at: https://www.mdpi.com/article/10.3390/jcm15020419/s1, Table S1. Odds ratios of ultrasonographic features associated with thyroid malignancy.
