# Supplementary material for: Beyond Size: Integrating Ultrasonographic Features and FNAB Cytology to Predict Thyroid Malignancy—A Retrospective, Single-Center Study
Source: J Clin Med. 2026 Jan 6;15(2):419. doi: 10.3390/jcm15020419 (PMC12841862; doi:10.3390/jcm15020419)
Supplement: Supplementary file 1 [file jcm-15-00419-s001.zip › jcm-3992646-supplementary.pdf]

**Supplementary Table S1.** Odds ratios of ultrasonographic features associated with thyroid malignancy

| **Feature** | **Category** | **Odds Ratios** |
|---|---|---|
| Echogenicity | Hypoechoic | 4.64 |
| | Hyperechoic | 0.17 |
| | Mixed | 1.26 |
| Internal Structure | Solid | 1.83 |
| | Cystic | 0.49 |
| | Mixed | 0.80 |
| Calcification | None | 0.23 |
| | Microcalcification | 5.29 |
| | Macrocalcification | 2.13 |
| Vascularity | Hypovascular | 0.59 |
| | Hypervascular | 1.68 |
| Margins | Regular | 0.23 |
| | Irregular | 4.43 |
